# Evaluation of lockdown effect on SARS-CoV-2 dynamics through viral genome quantification in waste water, Greater Paris, France, 5 March to 23 April 2020

**DOI:** 10.2807/1560-7917.ES.2020.25.50.2000776

**Published:** 2020-12-17

**Authors:** S Wurtzer, V Marechal, JM Mouchel, Y Maday, R Teyssou, E Richard, JL Almayrac, L Moulin

**Affiliations:** 1 Eau de Paris, R&D Laboratory, DRDQE Ivry/Seine, France; 2 Sorbonne Université, INSERM, Centre de Recherche Saint-Antoine2, Paris, France; 3 These authors are co-founders of the COVID-IA/PANDEMIA initiative; 4 Sorbonne Université, CNRS, EPHE, UMR 7619 Metis, , e-LTER Zone Atelier Seine, Paris, France; 5 Sorbonne Université, CNRS, Université de Paris, Laboratoire Jacques-Louis Lions (LJLL), et Institut Universitaire de France, Paris, France; 6 Department of Virology, Institut de Recherche Biomédicale des Armées, Bretigny sur Orge, France; 7 SIAAP, Service process-laboratoire SIAAP site Seine Amont, Valenton, France

**Keywords:** SARS-CoV-2, COVID-19, lockdown, quantification, waste water

## Abstract

**Introduction:**

Severe acute respiratory syndrome coronavirus 2 (SARS-CoV-2) is the etiological agent of coronavirus disease (COVID-19). People infected with SARS-CoV-2 may exhibit no or mild non-specific symptoms; thus, they may contribute to silent circulation of the virus among humans. Since SARS-CoV-2 RNA can be detected in stool samples, monitoring SARS-CoV-2 RNA in waste water (WW) has been proposed as a complementary tool to investigate virus circulation in human populations.

**Aim:**

To test if the quantification of SARS-CoV-2 genomes in WW correlates with the number of symptomatic or non-symptomatic carriers.

**Method:**

We performed a time-course quantitative analysis of SARS-CoV-2 by RT-qPCR in raw WW samples collected from several major WW treatment plants in Greater Paris. The study period was 5 March to 23 April 2020, including the lockdown period in France (from 17 March).

**Results:**

We showed that the increase of genome units in raw WW accurately followed the increase of human COVID-19 cases observed at the regional level. Of note, the viral genome could be detected before the epidemic grew massively (around 8 March). Equally importantly, a marked decrease in the quantities of genome units was observed concomitantly with the reduction in the number of new COVID-19 cases, 29 days following the lockdown.

**Conclusion:**

This work suggests that a quantitative monitoring of SARS-CoV-2 genomes in WW could generate important additional information for improved monitoring of SARS-CoV-2 circulation at local or regional levels and emphasises the role of WW-based epidemiology.

## Introduction

Severe acute respiratory syndrome coronavirus 2 (SARS-CoV-2) is a positive-sense, single-stranded RNA virus of the *Coronaviridae* family and the aetiologic agent of coronavirus disease (COVID-19). As at 17 May 2020, there were more than 2.5 million COVID-19 cases worldwide and more than 180,000 total COVID-19–related deaths. In France, more than 160,000 COVID-19 cases were identified as at 22 April. Virus transmission is mainly associated with the projection of respiratory droplets, although contamination through aerosols, hands and surfaces is likely [1]. SARS-CoV-2 may cause severe complications, particularly in elderly people or those suffering from comorbidities such as diabetes, hypertension, obesity or acquired/iatrogenic immunosuppression.

SARS-CoV-2 infection may initiate in the upper and/or the lower respiratory tracts. However, similarly to severe acute respiratory syndrome coronavirus (SARS-CoV-1) [2] and Middle East respiratory syndrome coronavirus [3], SARS-CoV-2 genomes have been detected in blood and faeces [4-6]. This suggests a possible enteric phase of the infection, although isolation of infectious virus from faeces seems difficult [7]. Although viral genomes are frequently detected in symptomatic patients’ faeces, the presence of infectious virus particles is still debated [7]. Diarrhoea has been reported in COVID-19 cases [8]. Importantly, SARS-CoV-2 genomes could be detected in faeces several weeks after it could no longer be detected in oral swabs, suggesting that viral excretion may last longer in faeces than in oral secretions [9]. The presence of viral genomes in faeces may offer a new perspective for the monitoring of SARS-CoV-2 infection in a population. Notably, it also suggests that the virus could be transmitted by a faecal-oral route, a hypothesis that warrants careful examination [10].

Management of epidemics may require careful monitoring of the infected population by detecting the causative pathogen through widespread or targeted testing. Exceptional measures such as lockdowns rely on this kind of information. Investigating the proportion of people that have been infected through sero-epidemiological surveys is equally important. Antibodies against SARS-CoV-2 do not appear until weeks after initial infection [11,12] and the precise number of infected people is difficult to assess because of the lack of systematic, repeated screening of the population. This is further complicated by the proportion of infected people that exhibit only few or no symptoms, but who could still shed and silently transmit the virus [13-16]. Depending on screening kit availabilities and public health policies, testing strategies vary between countries, which may explain some discrepancies between worldwide data on numbers of COVID-19 cases and fatalities.

Estimating the proportion of infected individuals is essential to monitoring the COVID-19 epidemic’s spread and proposing adapted and effective control measures, such as partial or total lockdowns. France went into lockdown on 17 March, a decision that was expected to have a major impact on virus circulation, especially as asymptomatic carriers are considered to affect virus transmission. This decision was motivated by the urgent need to limit exposure for people who are at highest risk of developing severe forms of the disease [13,15,17].

Analysis of raw waste water (WW) collected at the inlets of waste water treatment plants (WWTPs) may provide integrated information on the level of infection in the human population that is connected to the WWTP. Notably, it may allow measurement and identification of pathogens or drugs that may be difficult to assess otherwise. Using this method, the European Monitoring Centre for Drugs and Drug Addiction follows drugs and their metabolites in the WW of several European cities [18]. In addition, previous works on human enteric viruses in urban rivers and in raw and treated WW have demonstrated that the presence of these viruses was directly linked to the state of epidemics in the populations in the catchment areas [19,20]. This strongly argues for close monitoring of viruses shed in faeces in WW as an innovative and complementary tool for investigating human epidemics.

Enveloped viruses like coronaviruses are expected to be more sensitive to chemical or physical agents than the non-enveloped viruses that are usually tracked in WW and environmental waters. As at the beginning of the study period, there was still little information on the persistence of coronaviruses in water and most of our knowledge was inferred from experiments made on surrogate viruses. Early data suggest that infectious SARS-CoV-2 is able to persist in various environmental conditions, surviving 3.5 half-life days in the air and 7 days on some surfaces, and undergoing no observable decay at pH 3–10 for 1 hour [8,21]. Previous studies on SARS-CoV-1 demonstrated persistence of virus particles under an infectious form of > 20 days at 4°C (even in WW) and a persistence of at least 1 or 2 days at summer temperatures [2,22,23].

These results have led us and others to suggest that the detection of SARS-CoV-2 genomes in WW could provide an early and global tool to monitor virus circulation in addition to human epidemiological data [24-26]. A first publication underlined the putative benefits of a qualitative approach to monitoring SARS-CoV-2 in WW [26]. Another study used quantitative measurements of viral genomes, but the survey only started at the peak of the epidemic [27]. Here, we used a specific reverse-transcription quantitative PCR (RT-qPCR) method to precisely quantify SARS-CoV-2 genome equivalents in raw WW of the Greater Paris area. We favoured a procedure that concentrated viral genomes by ultracentrifugation, in contrast to other groups that used filtration methods. In our experience, ultracentrifugation of WW samples was easier to perform and provided equivalent or better virus recovery rate than filtration procedures. A 2-month survey, including the lockdown period in France, allowed us to evaluate the dynamics of viral genomes.

## Methods

### Sample collection

Three WWTPs, linked with 3,000,000 inhabitants of Greater Paris and processing almost 600,000 cubic meters of WW per day, were sampled since the start of the epidemic on 5 March 2020.

Average daily samples (according to NF T 90-90-523-2) consisting of 100 mL were taken by automated samplers. Sampling was based on flow rate and started at 7:00, finishing at D+1, 7:00. Samples were taken by suction using a PVC pipe with an ascensional speed higher than 0.8 m/s, and were collected in a refrigerated polyethylene tank at 5°C (+/- 3°C). The final collected volume was between 8.7 L and 14 L. Samples were then carefully homogenised and distributed in a 2 L polyethylene bottle. They were transported to the laboratory at 4°C and were processed less than 24 hours after sampling. A map of the area served by the sampled WWTPs is indicated in the Supplementary Figure.

### Concentration methods

Samples were homogenised, then 11 mL were centrifugated at 200,000 x g for 1 hour at 4°C using a XPN80 (Coulter Beckman, Fullerton, United States (US)) ultracentrifuge equipped with a swing rotor (SW41Ti). Viral pellets were resuspended in 200 μL of PBS 1X buffer. The viral concentrate was lysed and extracted using PowerFecal Pro kit (QIAGEN, Hilden, Germany) on a QIAsymphony automated extractor (QIAGEN, Hinden, Germany ), according to a modified manufacturer’s protocol using a larger volume of samples. Extracted nucleic acids were filtered through OneStep PCR inhibitor removal kit (Zymoresearch, Irvine, US), according to the manufacturer’s instructions.

### Molecular detection method

The RT-qPCR primers and PCR conditions used herein have been previously described [28]. The amplification was done using Fast virus 1-step Master mix 4x (Lifetechnologies, Carlsbad, US) with oligonucleotide concentrations recommended previously. Detection and quantification were carried on the E gene by RT-qPCR. Positive results were confirmed by amplification of a region located within the gene encoding for the viral RNA-dependent RNA polymerase (RdRp). An internal positive control (IPC) was added to evaluate the presence of residual inhibitors. The IPC consists in a plasmid-containing, beta-acting gene flanked by enterovirus-specific primers [29]. The detection limit was estimated to be around 10^3^ genome units per liter (GU/L) of raw WW.

The quantification was performed using a standard curve based on synthetic oligonucleotide corresponding to the full-length amplicon on the E gene (SARS-CoV-2 Wuhan-Hu isolate sequence NC_045512.2). Amplification reaction and fluorescence detection were performed on Viaa7 Real Time PCR system (Lifetechnologies).

### Epidemiological data collection and modelling of viral RNA excretion

Since the real number of infected people is unknown, we attempted to infer the estimated number of patients excreting the virus from published data on viral RNA concentration in faeces. Wölfel et al. measured the daily amount of vRNA in stool swab samples for a few patients [7] and the daily number of patients that consulted emergency departments in Greater Paris and were diagnosed with COVID-19 was published [30] from the very beginning of the epidemic. These two sets of information were used to produce an estimate of viral excretion for infected people. In this model, we assumed that people with symptoms would be admitted to the hospital 2 days after symptom onset on average, and that at any moment the number of patients with severe disease requiring hospitalisation represents a constant fraction of the total number of infected people. Accepting the above hypothesis, the convolution of two data sets (i.e. number of consulting patients and model of excretion) is an emission-proxy, proportional to the total amount of viral RNA shed in faeces in a given population.

## Results

The inlets of three major WWTPs of Greater Paris were sampled from 5 March to 23 April 2020. All processed samples tested positive for the presence of SARS-CoV-2 genomes, as assessed by RT-qPCR on the viral E gene. All positive samples were confirmed by RT-qPCR on the viral RdRp gene (Figure, Panel A).

Briefly, the concentration of vRNA in raw WW was around 5.10^4^ GU/L in samples collected from 4 March (7:00) to 5 March (7:00).

The time-course monitoring of viral load in WW displayed a strong increase, from 5.10^4^ GU/L on average in all WWTPs on 5 March to 3.10^6^ GU/L on 9 April, an almost 2-log increase. A peak observed on 9 April was followed by a marked decrease in the following days (1-log reduction).

The COVID-19 epidemic in Greater Paris was captured by various indicators, such as the daily total number of COVID-19 cases treated in regional hospitals, the daily increase in hospitalised patients and the daily number of COVID-19–related deaths (Figure, Panel B).

**Figure:**
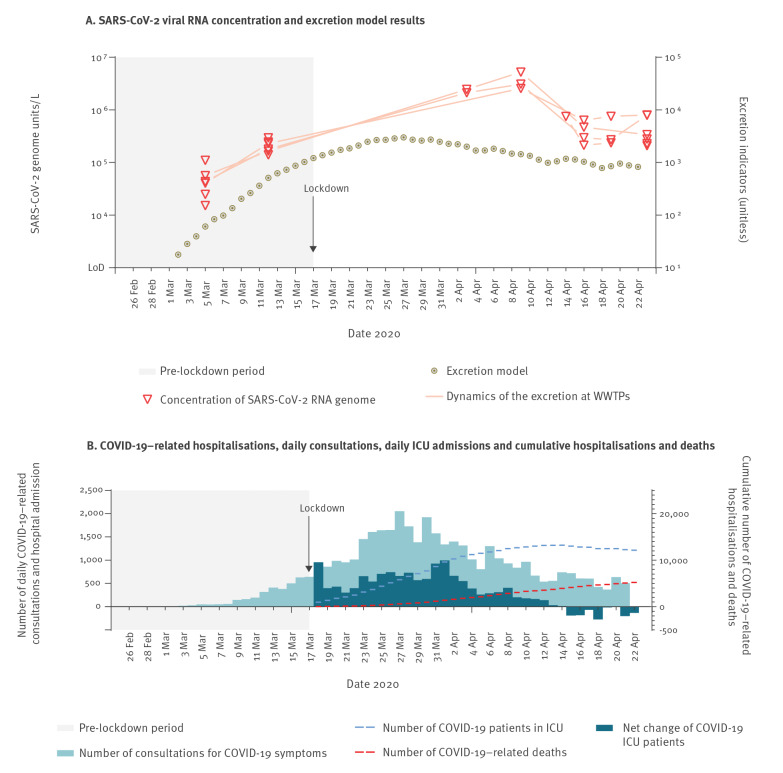
(A) Quantification of SARS-CoV-2 in waste water samples and (B) COVID-19–related hospitalisations, daily consultations, daily ICU admissions and cumulative hospitalisations and deaths, Greater Paris, France, 5 March–23 April 2020

COVID-19: coronavirus disease; ICU: intensive care unit; SARS-CoV-2: severe acute respiratory syndrome coronavirus 2; WWTPs: waste water treatment plants.

Panel A: The lines indicating the ‘dynamics of the excretion at WWTPs’ refers to three WWTPs, linked with 3,000,000 inhabitants of Greater Paris and processing almost 600,000 cubic meters of waste water per day, that were sampled during the study period.

Panel B: The net change of COVID-19 ICU patients is the difference between the number of COVID-19 patients in ICU on a given day, compared with the number of COVID-19 patients in ICU on the previous day.

On 4 March, less than 10 hospitalised COVID-19 cases were reported in the catchment areas of the studied WWTPs and only 635 were reported for the whole country. For Greater Paris more specifically, 64 confirmed cases were reported on that date (out of a total of more than 12 million inhabitants) and no deaths were reported. This information indicated that the COVID-19 epidemic was at an early stage in the Greater Paris area.

Based on these epidemiological data and published data on virus shedding quantity and delay, an estimated indicator of the viral load excreted by those infected with SARS-CoV-2 in Greater Paris was calculated and compared to the viral load in WW. The shape of the concentration curve was reminiscent of the disease dynamics at the regional level, i.e. the number of daily positive cases, with an 8-day temporal shift.

## Discussion

Our study, conducted early in the COVID-19 pandemic in France, demonstrated that a quantitative detection of SARS-CoV-2 in WW could reflect the circulation of the virus in the human population in Greater Paris. Since similar results were obtained from three independent and distant WWTPs—with striking similarities—the course over time is likely to be a direct reflection of SARS-CoV-2 dynamics in the populations connected to these WWTPs. It should be noted that a lockdown was put in effect on 17 March 2020, thereby limiting inhabitants’ movements. Importantly, no significant rainfall that could have had an impact on virus concentration in WW was recorded after 2 April in the region during the study period. More surprisingly, the decrease of the viral RNA concentration stopped after 7 days, and the virus concentration was stable thereafter. While this plateau is intriguing, the emission-proxy suggests that it can partly be explained by the duration of the virus shedding period, and several hypotheses can be made.

First, one may suggest that many infected people were still shedding infectious or non- infectious viruses in their faeces, whereas RNA and the virus were not present anymore in respiratory secretions. This hypothesis has recently been confirmed in Chinese patients with longer periods of excretion (more than a month) than initially reported by Wölfel et al. [9]. Second, lockdown was partial, as some specific workers were allowed to pursue essential activities that were not compatible with working at home. These people were usually not considered to be at risk of severe infection (i.e. more pauci-symptomatic cases), but they may promote virus circulation at a low level—notably in their household—if they do not strictly respect guidelines regarding handwashing, physical distancing and mask wearing. Third, one may speculate that the lockdown was not respected by a few people that maintained virus circulation at a low but relevant level. Virus surveillance in the same WWTPs will likely provide some answers in the near future.

The observed delay between epidemiological curves in humans compared to viral RNA quantification in WW is probably due to several parameters. This may include the effective number of infected people, the timing and temporal kinetics of viral RNA shedding in faeces and other causes that are still to be investigated. Nevertheless, our data are in very good agreement with epidemiological parameters, such as the number of confirmed COVID-19 cases or our excretion model. To that respect, we note that our study provides strong indirect vidence for a relevant reduction of virus transmission in response to a lockdown. According to our results, the number of people infected by SARS-CoV-2 is likely underestimated when based on individual testing, especially during the onset of the COVID-19 pandemic, when a limited availability of virological tests did not allow for extensive testing.

The highest concentration of SARS-CoV-2 genomes found in our study was about 2.5 10^6^ UG/L As a comparison, the concentration of human enteric virus in raw WW is around 10^6^ UG/L [19].

Epidemiological investigations conducted on the Diamond Princess cruise ship suggested that less than 20% of SARS-CoV-2–infected people were asymptomatic [14]. Since the Diamond Princess cases were reported to exhibit moderate non-specific symptoms including fever, headache, body aches, intense tiredness and/or dry cough. However, infected people can replicate SARS-CoV-2 for a few days before the onset of symptoms and up to several days after recovery [3,9,31]. Another extensive study based on the Icelandic population showed that 43% of SARS-CoV-2–positive people did not report any symptoms [32]. In this context, a number of those infected may silently spread the virus and contaminate others. This led us to suggest that the contamination of raw WW may occur before the appearance of clinical cases. The evolution of SARS-CoV-2 RNA viral load in WW was in reasonable agreement with the dynamics of the first wave of the COVID-19 epidemic in Greater Paris, which is also in agreement with the excretion model proposed here. Therefore, complementary studies are required to precisely monitor viral load in faeces over time in both symptomatic and non-symptomatic infections, including in children. To our knowledge, this is the first study demonstrating that the quantitative monitoring of SARS-CoV-2 in raw WW is a time-related, relevant indicator of the evolution of the viral status of a population linked to a sewage network. This quantitative approach supported observation of the dynamics of the epidemic in Greater Paris and the impact of government measures, such as the lockdown.

These data, if carefully utilised, could help to describe the proportion of people that are infected by SARS-CoV-2 and are excreting viral RNA during monitored pandemic events and could allow for calculation of a population’s possible exposure to new infections, especially at the local level. The results underline that important information could be obtained from epidemiological monitoring of WW, such as identifying the early phase of epidemics, the onset of virus circulation, the evolution of infections, the impact of lockdowns or the effectiveness of barrier measures in a local area. To our knowledge, this is the first real-time integrated survey of SARS-CoV-2 circulation during a lockdown period

### Conclusions

Our results strongly argue for the use of quantitative surveillance of SARS-CoV-2 genomes in urban WW. Further, long-time conservation of WW samples in a dedicated, regional WW bank would allow for retrospective investigation of a pathogen’s circulation. Additionally, WW surveillance may provide an alternative and possibly early tool to detect pathogens in populations in an integrated way when investigations in humans are difficult to conduct for logistical, ethical or economical reasons, including in low-resource countries affected by the COVID-19 pandemic.

## Supplementary Data

900000 Supplementary Figure
