# Supplementary material for: Evaluation of lockdown effect on SARS-CoV-2 dynamics through viral genome quantification in waste water, Greater Paris, France, 5 March to 23 April 2020
Source: Euro Surveill. 2020 Dec 17;25(50):2000776. doi: 10.2807/1560-7917.ES.2020.25.50.2000776 (PMC7812418; doi:10.2807/1560-7917.ES.2020.25.50.2000776)

This supplementary material is hosted by *Eurosurveillance* as supporting information alongside the article *Evaluation of lockdown effect on SARS-CoV-2 dynamics through viral genome quantification in waste water, Greater Paris, France, 5 March to 23 April 2020* on behalf of the authors who remain responsible for the accuracy and appropriateness of the content. The same standards for ethics, copyright, attributions and permissions as for the article apply. Supplements are not edited by *Eurosurveillance* and the journal is not responsible for the maintenance of any links or email addresses provided therein.

**Supplementary Figure. Area covered by the station monitored in this work (coloured cities) in the whole Greater Paris**

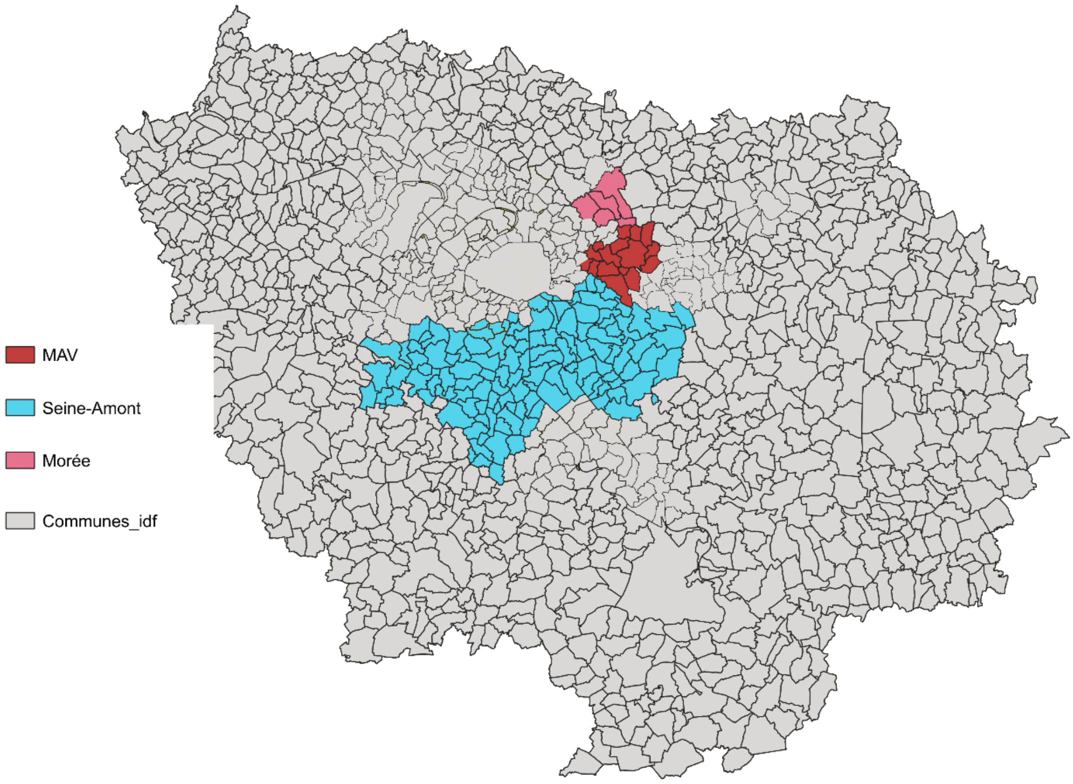

Supplement: Supplementary Figure [file 2000776_SupplementaryFigure.pdf]
